# Body composition–based nutritional status during neoadjuvant chemotherapy and its association with relative dose intensity and hematologic toxicity in patients with gastric cancer

**DOI:** 10.3389/fnut.2026.1753568

**Published:** 2026-04-16

**Authors:** Guoqing Shi, Penghui Liu, Na Li, Yan Dong, Tianyu Gao, Peng Du, Jiwu Guo, Jie Mao

**Affiliations:** 1 The Second Clinical Medical College, Lanzhou University, Lanzhou, Gansu, China; 2 Lanzhou University Second Hospital, Lanzhou, Gansu, China

**Keywords:** body composition, gastric cancer, hematological toxicity, neoadjuvant therapy, relative dose intensity

## Abstract

**Objective:**

This study investigated changes in body composition before and after neoadjuvant therapy in patients with gastric cancer and explored the relationships between these changes, relative dose intensity (RDI), and hematological toxicity.

**Methods:**

Body composition parameters (muscle mass, skeletal muscle mass, body fat mass, visceral fat area, and water content) were measured via an InBody 720 analyzer before and after treatment. The primary outcome was a reduced RDI (<85%), and the secondary outcome was Grade 2 or 3 hematological toxicity.

**Results:**

Patients with a reduced RDI (<85%) presented significant decreases in muscle mass, skeletal muscle mass, and total body water content after therapy and had longer treatment duration. Greater muscle mass, skeletal muscle mass, and total body water content were associated with lower risks of reduced RDI [OR: 0.808 (0.697–0.938)], [OR: 0.699 (0.545–0.897)], and [OR: 0.759 (0.626–0.920)], respectively. Grade 2 thrombocytopenia was associated with reduced muscle mass and total body water, as well as increased body fat mass and visceral fat area. In further regression analyses, greater skeletal muscle mass was associated with a lower risk of grade 2 thrombocytopenia [OR: 0.657 (0.434–0.996)].

**Conclusion:**

Higher muscle mass and total body water were associated with a lower risk of reduced RDI, whereas body fat mass showed no significant association. Regarding hematologic toxicity, grade 2 thrombocytopenia was associated with adverse changes in body composition, and greater skeletal muscle mass was associated with a lower risk in the adjusted analysis.

## 1 Introduction

Gastric cancer is a global health problem. In 2022, there were over 968,000 new cases of stomach cancer and nearly 660,000 deaths worldwide. Although the incidence and mortality of gastric cancer have declined globally over the past 50 years, it remains the fifth leading cause of cancer-related deaths (1–2). Although surgical resection is key to achieving a cure, even for relatively early-stage tumors, surgery alone may be associated with lower survival rates. A multimodal approach, combining neoadjuvant therapy, adjuvant therapy, or perioperative chemotherapy with surgical treatment, has been shown to improve survival rates. Neoadjuvant therapy is recommended in the guidelines of multiple regions and countries for locally advanced gastric cancer (3–4). For patients undergoing treatment, the dosage of chemotherapy drugs is calculated based on the body surface area, which is determined by their height and weight (5). However, body surface area may have limitations when it comes to factors that can affect drug metabolism, such as muscle mass, fat, and water content. Additionally, the metabolism of a drug in the body can further influence its efficacy and side effects. Body composition—in terms of the amount and distribution of muscle and fat mass—has also been significantly related to chemotherapy toxicity and tolerance (6). Tumor patients often experience changes in body composition over time due to reduced food intake, increased metabolic demands, inflammation and catabolic reactions caused by the tumor, as well as side effects related to treatment. Therefore, integrating body composition assessment into the clinical evaluation of cancer patients becomes crucial (7). With the development of technology, methods for measuring body composition have become increasingly diverse, with common techniques including DXA, bioelectrical impedance analysis (BIA), CT, and other (8). Among them, bioelectrical impedance analysis has been proven to be a noninvasive and reliable method widely used to assess body components such as muscle, fat, water, inorganic salt, and protein (9–10). It can quickly measure muscle mass, skeletal muscle mass, body fat mass, visceral fat area, water content, and other parameters of the body. Previous studies have confirmed that sarcopenia and muscle fat infiltration are among the factors contributing to poor prognosis in gastric cancer patients (11). Additionally, the skeletal muscle index, total body fat mass, and other parameters are predictive indicators of chemotherapy toxicities in patients with gastrointestinal cancers (7, 12). Although muscle loss as a prognostic factor has been associated with relative dose intensity (RDI) in gastric cancer (13), the direct relationship between baseline body composition and RDI has not been well established. Relative dose intensity (RDI), which reflects dose reductions and treatment delays, is an important indicator of chemotherapy delivery. Although the optimal RDI threshold may vary according to cancer type, stage, and regimen, reduced dose intensity has been associated with poorer outcomes in gastric cancer. In particular, a study in patients with stage II/III gastric cancer receiving adjuvant S-1 chemotherapy showed that insufficient RDI was associated with poorer survival outcomes. In the present study, we used RDI < 85% as a pragmatic definition of reduced dose intensity (14). Some studies suggested that a reduced RDI (Relative Dose Intensity) not only diminishes the effectiveness of chemotherapy but also may lead to poor prognosis in cancer patients (15–16). Adverse events during chemotherapy cycles are the primary reasons for the reduction in RDI. Hematologic toxicities are one of the most common adverse reactions, often affecting treatment efficacy and sometimes leading to premature treatment termination (17).

Given the limitations of previous research, we designed this study to assess changes in various body composition parameters (muscle, fat, and water content) before and after neoadjuvant therapy in gastric cancer patients, and to explore the relationships between these changes, reduced treatment RDI, and hematologic toxicities.

## 2 Materials and methods

### 2.1 Study population

**Figure 1:**
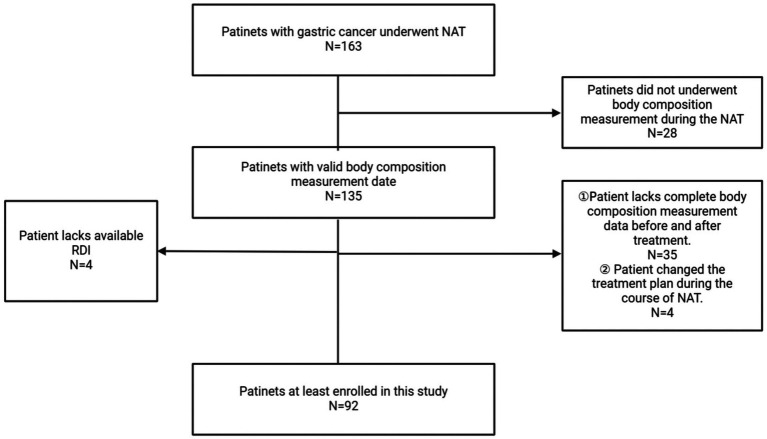
Flowchart of the study.

This study initially retrospectively included 136 patients who were diagnosed with gastric malignant tumors and received neoadjuvant therapy at the Second Hospital of Lanzhou University from May 2021 to January 2024. This study adhered to the Declaration of Helsinki and was approved by the Ethics Committee of the Second Hospital of Lanzhou University. Among the initially screened patients, 28 were excluded because at least one body composition measurement obtained before or after neoadjuvant therapy was invalid or not analyzable. Subsequently, 35 additional patients were excluded because complete paired body composition data before and after treatment were unavailable. In addition, 4 patients were excluded because of changes in treatment regimen, and 4 were excluded because useful RDI data were unavailable. Finally, 92 patients were included in this study (Figure 1). Inclusion criteria were as follows: ① Age ≥18 years and pathologically confirmed diagnosis of gastric malignant tumors; ② Complete and valid body composition measurement data before and after neoadjuvant therapy; ③ Neoadjuvant therapy was completed at a single institution from the time of diagnosis to the next treatment. Exclusion criteria included: ① Presence of limb dysfunction; ② Use of medications that could affect body composition, such as diuretics, during body composition measurement; ③ Coexisting psychiatric or consciousness disorders; ④ Change in treatment regimen during therapy; ⑤ Pregnancy or breastfeeding; ⑥ Lack of valid RDI or laboratory test results.

This study primarily included patients who received one of the following three treatment regimens:

① SOX (Oxaliplatin: 130 mg/m^2^ + S-1:40 mg/50 mg/60 mg/75 mg (Based on body surface area)) + PD-1 inhibitor;

② XELOX (Capecitabine: 1250 mg/m^2^ + Oxaliplatin:130 mg/m^2^) + PD-1 inhibitor;

③ FLOT (Oxaliplatin: 85 mg/m^2^ + Docetaxel: 60 mg/m^2^ + 5-Fluorouracil: 2600 mg/m^2^ + Leucovorin: 200 mg/m^2^) + PD-1 inhibitor.

The PD-1 inhibitors used were Sintilimab or Camrelizumab.

### 2.2 Body composition

The body composition indicators in this study included muscle mass, skeletal muscle mass, body fat mass, visceral fat area, and water content. Body composition parameters were measured using an InBody 720 body composition analyzer (Biospace Co., Ltd., Seoul, Korea), a multi-frequency bioelectrical impedance analyzer with eight-point tactile electrodes, before and after NAT. The measurement was performed in an operational environment with air temperature of 20–25 °C and humidity of 30–75%. Every measurement was performed in compliance with the manufacturer’s user instructions. Before each measurement, patients emptied their stomach, removed shoes and hats, and stood calmly with their arms naturally hanging by their sides (18). All measurements and adjustments were performed by nurses who had received standardized training in the proper operating procedures. Height and weight were measured and recorded using standard methods. BMI was calculated as Weight (kg)/Height (m)^2^, and BSA was calculated as [Height (cm) × Weight (kg)/3,600]. These variables were recorded as baseline anthropometric parameters. They were not the primary body composition exposures analyzed in the main regression models.

Changes in body composition were defined as the absolute difference between post-treatment and pre-treatment measurements (*Δ* = post-treatment value − pre-treatment value). Positive values indicated an increase during neoadjuvant therapy, whereas negative values indicated a decrease. In the regression analyses, body composition variables were entered as continuous change variables; therefore, “increase” refers to a greater positive absolute change in the corresponding parameter during treatment.

### 2.3 Outcomes

The primary outcome measure of this study was reduced relative dose intensity (RDI < 85%), which was defined as the average RDI for each patient throughout the entire neoadjuvant treatment period. The data was obtained from electronic medical records, collecting information on each treatment cycle, including the administration dates and doses of each drug. RDI was calculated for each drug separately, and then the average of all drugs was taken. The calculation formula is as follows (19):


$$\mathrm{RDI}=\frac{\left(\text{delivered total dose in}\;\mathrm{mg}/m^{2})/(\text{actual time to complete treatment}\right)}{\left(\text{planned total dose in}\;\mathrm{mg}/m^{2})/(\text{planned time to complete treatment}\right)}$$

The secondary outcome of this study was hematological toxicity induced by neoadjuvant therapy, including anemia, neutropenia, thrombocytopenia, and leukopenia. These hematological toxicities were classified according to Common Terminology Criteria for Adverse Events (CTCAE) version 5.0. In this study, we primarily focused on grade 2 (clinically significant) and grade 3 (severe) adverse events during the entire treatment period. Grade 2 anemia was defined as hemoglobin (HB) < 10 g/dL; grade 2 neutropenia as neutrophils (NEU) < 1.5 × 10^9^/L; grade 2 thrombocytopenia as platelets (PLT) < 75 × 10^9^/L; grade 2 leukopenia as white blood cells (WBC) < 3.0 × 10^9^/L. Grade 3 anemia was defined as HB < 8 g/dL; grade 3 neutropenia as NEU < 1.0 × 10^9^/L; grade 3 thrombocytopenia as PLT < 50 × 10^9^/L; and grade 3 leukopenia as WBC < 2.0 × 10^9^/L (20). In cases where multiple grades of anemia, leukopenia, neutropenia, or thrombocytopenia were present, the highest grade was used as the basis for evaluation. Hematological test results during treatment were obtained through the HIS system.

### 2.4 Other data

The other patient data in this study include: age (years), gender (male, female), duration of neoadjuvant therapy (weeks), tumor location (gastric cardia, gastric body, pylorus), pathological stage (I & II, III & IV), pathological type (adenocarcinoma, adenocarcinoma + SIG, MUA), and Ki-67.

### 2.5 Statistical analysis

Descriptive statistics for continuous variables included the mean and standard deviation (SD) or median and interquartile range (IQR), while categorical variables were described using counts and percentages. Differences in baseline characteristics between patients with different RDI statuses (<85% or ≥85%) and the relationship between various body composition measurements and hematological toxicity during treatment were compared using Student’s t-test or the Mann–Whitney U test for continuous variables and the Chi-square (χ^2^) test for categorical variables. Subsequently, two separate binary logistic regression models were constructed. The first model evaluated the association between each 1-SD increase in the change in body composition and reduced RDI during neoadjuvant therapy. The second model evaluated the association between each 1-SD increase in the change in body composition and grade 2 thrombocytopenia during neoadjuvant therapy. Both models were adjusted for age, sex, height, treatment duration (weeks), treatment regimen, and tumor stage. All statistical analyses were performed using SPSS version 26.0. Figures were created using GraphPad Prism version 10.0. For all statistical results, a *p*-value <0.05 or a 95% confidence interval not including 1 (for odds ratios [OR]) was considered statistically significant.

## 3 Results

A total of 92 gastric cancer patients who received neoadjuvant therapy were included in the study. The majority of the patients were male (88.04%). The average age was 59 years, and the median duration of neoadjuvant therapy was approximately 16 weeks. The majority of tumors were located in the pyloric region (49.91%). About 76% of the patients were in pathological stages III-IV. In this study, FLOT+PD1 was the main regimen for neoadjuvant therapy (78.02%). In terms of body composition, the largest change observed before and after treatment was in visceral fat area (25.06%) (Table 1).

**Table 1:** Demographic and clinical of the study cohort.

Characteristic	Overall cohort (*N* = 92)
Gender (male), *n* (%)	81 (88.04)
Age (years), mean (SD)	59.26 (9.17)
Duration of neoadjuvant treatment (weeks), median (IQR)	16.00 (12.00, 17.00)
Tumor location, *n* (%)	
Cardia	33 (35.87)
Gastric body	14 (15.22)
Pylorus	45 (48.91)
Pathological stage, *n* (%)	
I&II	22 (24.18)
III&IV	69 (75.82)
Pathological type, *n* (%)	
Adenocarcinoma	55 (59.78)
Adenocarcinoma+SIG	5 (5.43)
MUA	4 (4.35)
Missing	28 (30.4)
Ki-67 (%), median (IQR)	70.00 (60.00,80.00)
Treatment regimen, *n* (%)	
FLOT+PD1	71 (78.02)
SOX+PD1	15 (16.48)
XELOX+PD1	5 (5.49)
SLM change (kg), mean (SD)	−0.41 (3.63)
BFM change (kg), mean (SD)	−1.09 (3.14)
SMM change (kg), mean (SD)	−0.24 (2.17)
VFA change (cm^2^), mean (SD)	−9.13 (25.06)
TBW change (L), mean (SD)	−0.36 (2.80)
BMI change (kg/m^2^), median (IQR)	−0.70 (-1.80, 0.18)

SLM, muscle mass; BFM, body fat mass; SMM, skeletal muscle mass; VFA, visceral fat area; TBW, total water content; BMI, body mass index; SIG, signet-ring cell carcinoma; MUA, mucinous adenocarcinoma Continuous variables were presented as mean (SD) or median (IQR) and categorical variables as number (%).

Additionally, we also investigated the body composition before and after treatment (Supplementary Table 1). Gender-specific changes in body composition were shown in Supplementary Table 2. Patients with reduced RDI (RDI < 85%) had longer treatment duration and less muscle mass, skeletal muscle mass, and total body water than those with normal RDI (Table 2).

**Table 2:** Patient characteristic among 92 patients with gastric cancer by RDI status (<85 vs. ≥ 85%).

Characteristic	RDI < 85% (*N* = 46)	RDI ≥ 85% (*N* = 46)	*p*-value
Age (years)	59.3 (9.58)	59.2 (8.85)	0.964
Gender			0.335
Male	39 (84.78)	42 (91.30)	
Female	7 (15.22)	4 (8.70)	
Duration of neoadjuvant treatment (weeks)	16.5 (11.8,19.0)	15.0 (11.8,17.0.0)	0.038
Tumor location			0.098
Cardia	18 (39.13)	15 (32.61)	
Gastric body	10 (21.74)	4 (8.70)	
Pylorus	18 (39.13)	27 (58.70)	
Pathological stage			
I&II	8 (17.78)	14 (30.43)	
III&IV	37 (82.22)	32 (69.57)	
Pathological type			0.975
Adenocarcinoma	28 (60.87)	27 (58.70)	
Adenocarcinoma+SIG	2 (4.35)	3 (6.52)	
MUA	2 (4.35)	2 (4.35)	
Missing	14 (30.43)	14 (30.43)	
Ki-67 (%)	70.0 (30.0,80.0)	70.0 (60.0, 80.0)	0.369
Treatment regimen			0.874
SOX+PD1	35 (77.78)	36 (78.26)	
XELOX+PD1	7 (15.56)	8 (17.39)	
FLOT+PD1	3 (6.67)	2 (4.35)	
SLM change (kg)	−1.34 (3.45)	0.53 (3.60)	0.013
BFM change (kg)	−1.20 (3.41)	−0.99 (2.89)	0.755
SMM change (kg)	−0.80 (2.06)	0.32 (2.15)	0.012
VFA change (cm^2^)	−9.20 (25.99)	−9.07 (24.39)	0.980
TBW change (L)	−1.09 (2.67)	0.36 (2.77)	0.042
BMI change (kg/m^2^)	−0.95 (−2.1, -0.3)	−0.40 (−1.6,0.9)	0.119

SLM, muscle mass; BFM, body fat mass; SMM, skeletal muscle mass; VFA, visceral fat area; TBW, total water content; BMI, body mass index; SIG, signet-ring cell carcinoma; MUA, mucinous adenocarcinoma.

**Figure 2:**
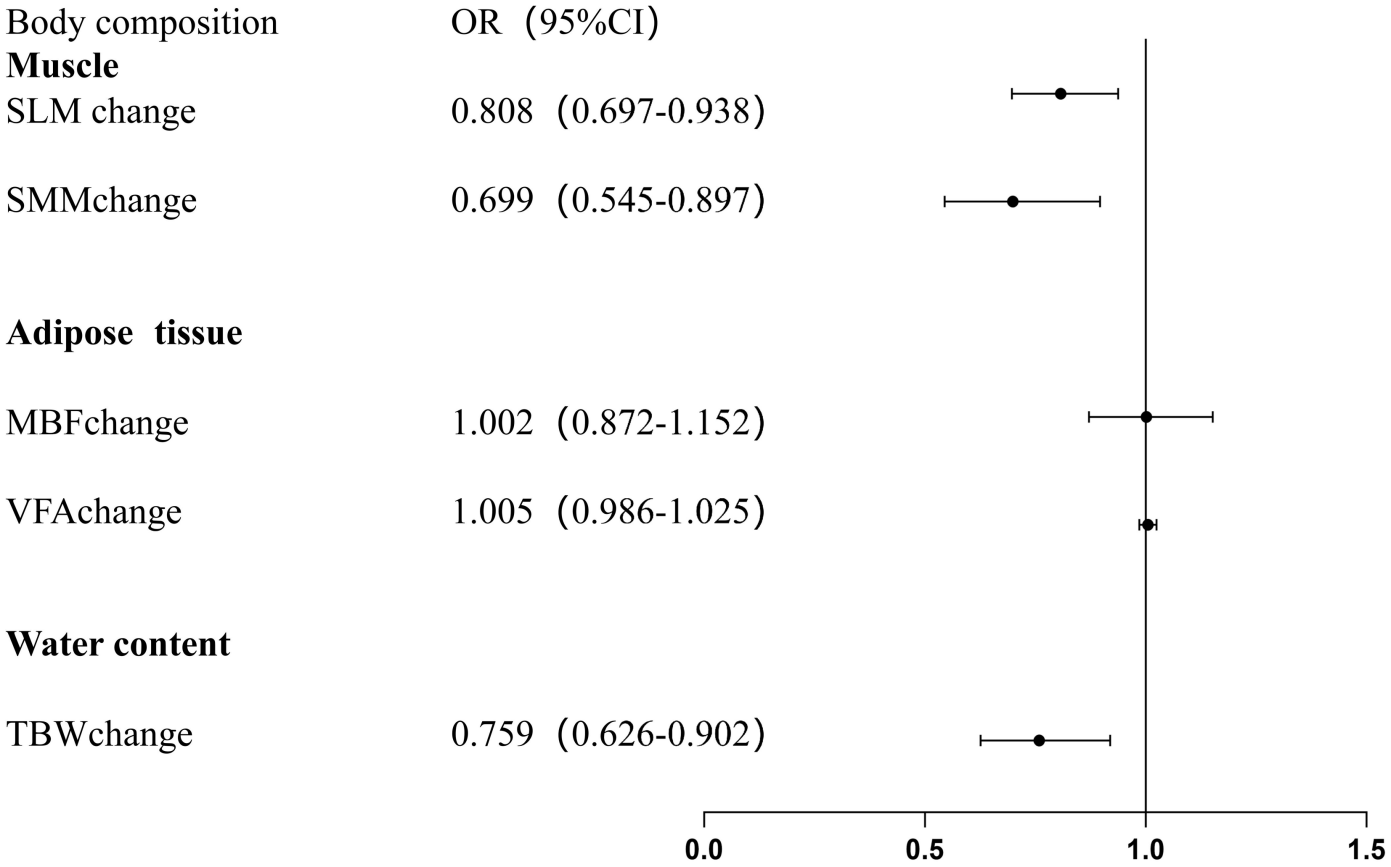
Adjusted associations between changes in body composition and reduced relative dose intensity (RDI < 85%) during neoadjuvant therapy.

For each 1-SD increase in muscle mass[OR:0.875 (0.754–0.973)], skeletal muscle mass[OR:0.771 (0.623–0.954)], and total body water content[OR:0.817 (0.693–0.964)], the risk of having a reduced RDI (RDI < 85%) decreased. This relationship remained significant even after adjusting for age, gender, duration of treatment, pathological stage, treatment regimen and height. However, no such relationship was observed between body fat mass, visceral fat area, and RDI (Table 3; Figure 2).

**Table 3:** The unadjusted and adjusted associations of body composition measurement with the odds ratios for reduced RDI (RDI<85%).

Body composition	Unadjusted model OR (95%CI)	*p*-value	Adjusted model OR (95%CI)	*p*-value
Muscle				
SLM change (kg)	0.857 (0.754–0.973)	0.017	0.808 (0.697–0.938)	0.005
SMM change (kg)	0.771 (0.623–0.954)	0.017	0.699 (0.545–0.897)	0.005
Adipose tissue				
BFM change (kg)	0.979 (0.859–1.116)	0.752	1.002 (0.872–1.152)	0.978
VFA change (cm^2^)	1.000 (0.984–1.016)	0.980	1.005 (0.986–1.025)	0.598
Water content				
TBW change (L)	0.817 (0.693–0.964)	0.016	0.759 (0.626–0.920)	0.005

SLM, muscle mass; BFM, body fat mass; SMM, skeletal muscle mass; VFA, visceral fat area; TBW, total water content.

Adjusted for age, gender, duration of treatment, pathological stage, treatment regimen and height.

**Figure 3:**
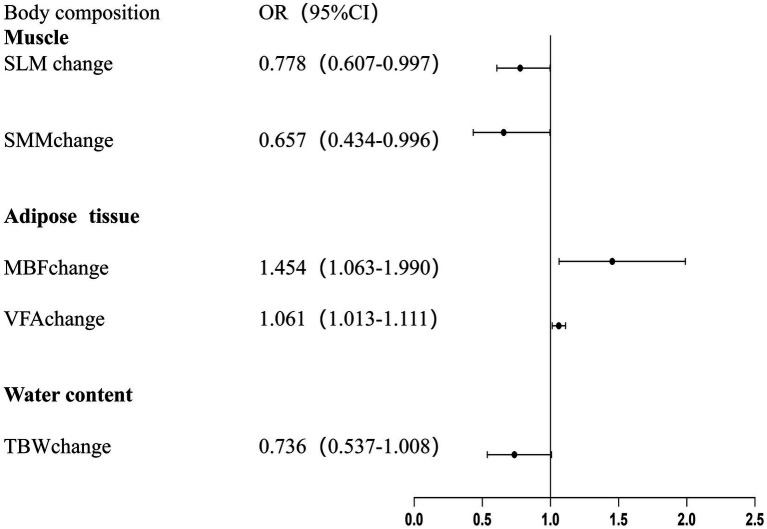
Adjusted associations between changes in body composition and grade 2 thrombocytopenia during neoadjuvant therapy.

Supplementary Table 3 presents the relationship between changes in body composition during neoadjuvant therapy and grade 2 hematologic toxicities, whereas Supplementary Table 4 presents the relationship with grade 3 hematologic toxicities. For grade 2 toxic reactions, we observed that changes in all body composition parameters were associated with grade 2 thrombocytopenia. In patients with grade 2 thrombocytopenia, there was a significant decrease in muscle mass, skeletal muscle mass, and total body water, while body fat mass and visceral fat area significantly increased. In particular, the average increase in visceral fat area was 7.90 cm^2^ in patients with grade 2 thrombocytopenia. However, for other grade 2 toxicities, such as anemia, neutropenia, and leukopenia, no significant differences in body composition changes were observed between patients with and without these toxicities. Similarly, no significant differences in body composition changes were found for grade 3 hematologic toxicities. Figure 2 presents the adjusted results of Regression Model 1 for reduced RDI (RDI < 85%). Figure 3 presents the adjusted results of Regression Model 2 for grade 2 thrombocytopenia.

Since our previous analyses showed that changes in body composition were significantly associated only with grade 2 thrombocytopenia, we further explored the relationship between body composition and the risk of grade 2 thrombocytopenia. In unadjusted analyses, a 1-SD increase in muscle mass was associated with a lower risk of grade 2 thrombocytopenia, whereas 1-SD increases in body fat mass and visceral fat area were associated with higher risks. After adjustment for age, sex, treatment duration, treatment regimen, and height, only skeletal muscle mass remained significantly associated with a lower risk of grade 2 thrombocytopenia (Table 4; Figure 3).

**Table 4:** The unadjusted and adjusted associations of body composition measurement with the Odds ratios for grade 2 thrombocytopenia.

Body composition	Unadjusted model OR (95%CI)	*p*-value	Adjusted model OR (95%CI)	*p*-value
Muscle				
SLM change (kg)	0.836 (0.698–1.000)	0.05	0.778 (0.607–0.997)	0.047
SMM change (kg)	0.742 (0.549–1.003)	0.053	0.657 (0.434–0.996)	0.048
Adipose tissue				
BFM change (kg)	1.392 (1.059–1.832)	0.018	1.454 (1.063–1.990)	0.019
VFA change (cm^2^)	1.050 (1.009–1.902)	0.017	1.061 (1.013–1.111)	0.012
Water content				
TBW change (L)	0.802 (0.636–1.011)	0.062	0.736 (0.537–1.008)	0.056

SLM, muscle mass; BFM, body fat mass; SMM, skeletal muscle mass; VFA, visceral fat area; TBW, total water content.

Adjusted for age, gender, duration of treatment, treatment regimen and height.

## 4 Discussion

This study described the relationship between changes in body composition during the NAT period in gastric cancer patients and RDI (Relative Dose Intensity) as well as hematologic adverse reactions. We found that during neoadjuvant therapy, an increase in muscle mass (muscle quantity, skeletal muscle mass) and water content (TBW) was associated with a reduced risk of experiencing a decreased RDI. However, no such relationship was observed for body fat mass. For hematologic adverse reactions during treatment, we found that grade 2 thrombocytopenia was associated with reduced muscle mass and increased adiposity during neoadjuvant therapy. In the adjusted analysis, greater skeletal muscle mass remained associated with a lower risk of this condition.

Relative dose intensity (RDI) is an indicator of chemotherapy delivery, calculated as the ratio of the actual chemotherapy dose administered to the initial prescribed dose, taking into account both dose intensity and treatment duration. It serves as a measure of chemotherapy adherence and is associated with cancer mortality. Although the optimal RDI threshold may vary according to cancer type, stage, and regimen, reduced dose intensity has been associated with poorer outcomes in gastric cancer. In the present study, we used RDI < 85% as a pragmatic definition of reduced dose intensity (21–22). A previous randomized trial on exercise and nutrition in women with breast cancer investigated the impact of exercise and a healthy diet on chemotherapy completion and pathological complete response, specifically examining their effects on relative dose intensity (RDI) (12). Research on the relationship between body composition and RDI is relatively scarce. One such study, conducted by En Cheng et al., explored the relationship between body composition and RDI in colorectal cancer patients. They utilized various body composition measurement techniques, including CT and DXA, to investigate this relationship. The study similarly found that higher muscle mass (pre-chemotherapy) was associated with a reduced risk of experiencing a decreased RDI [OR:0.56 (0.38–0.81)]. However, to the best of our knowledge, there have been very few studies on the relationship between changes in body composition and RDI during neoadjuvant therapy in gastric cancer patients. Yusuke Kono et al. conducted a study on the relationship between preoperative skeletal muscle volume and the S-1 dose intensity in elderly stage II/III gastric cancer patients undergoing adjuvant chemotherapy. They found that a higher preoperative skeletal muscle index (SMI) significantly increased the RDI of S-1 adjuvant chemotherapy by 62% (*p* = 0.03). In contrast, in the low SMI group, more patients experienced S-1-induced nausea (*p* = 0.03) and had to discontinue chemotherapy due to adverse events (*p* = 0.02) (23). However, no studies have reported on the relationship between body fat mass, total body water (TBW), and RDI. Although we did not find a significant risk relationship between body fat mass and decreased RDI, for total body water (TBW), we observed results similar to those for muscle tissue. Total body water (TBW) is an important physiological parameter, and changes in TBW are significant for maintaining fluid balance in the body. It also plays a crucial role in the diagnosis and monitoring of certain diseases (24–25). Since skeletal muscle contains a large amount of water, accounting for approximately 75% of its total mass, a reduction in total body water (TBW) can impair the absorption of nutrients by muscle tissue, increase friction between muscle fibers, and negatively impact muscle elasticity, contraction, and overall function (26). This may further explain the consistency between TBW and muscle tissue results. Regarding the relationship between body water content and RDI, Kenji Yamaoka et al. conducted a study on the ratio of intracellular water to total body water (ICW/TBW) and its impact on dose intensity in patients with advanced hepatocellular carcinoma treated with lenvatinib. They found that a ratio of extracellular water to total body water (ECW/TBW) < 0.400 at the start of treatment was associated with an RDI > 50% ([OR], 6.94; 95% [CI], 1.00–48.00; *p* = 0.049) (27). There are relatively few studies specifically focusing on the relationship between total body water (TBW) and treatment outcomes in cancer therapy.

It should also be acknowledged that the relationship between muscle-related body composition measures and reduced RDI may be bidirectional. On the one hand, better preservation of muscle mass may improve treatment tolerance and reduce the likelihood of dose reduction or delay. On the other hand, patients who are more tolerant of neoadjuvant therapy and experience fewer adverse events may be more likely to maintain their nutritional intake, physical activity, and muscle mass during treatment. Therefore, the observed association should not be interpreted as a strictly one-way causal relationship.

Due to the narrow therapeutic window of most chemotherapy drugs, individualized assessment of chemotherapy dosing is crucial. Many chemotherapy drugs are hydrophilic, and as mentioned earlier, skeletal muscle is a highly water-rich organ (approximately 75%). Additionally, muscle tissue has been shown to be closely linked to the body’s immune function. Excessive body fat mass, on the other hand, can impair immune recovery. Therefore, closely monitoring body composition, including muscle and fat, during treatment is vital for preventing treatment-related risks (28–29). When cancer patients receiving drug treatment experience adverse events such as anemia, neutropenia, and thrombocytopenia, these events can impact clinical responses, including drug dosage and treatment duration, and can ultimately reduce the effectiveness of the treatment (30).

Regarding the relationship between body composition and adverse events during treatment in gastric cancer patients, Wing-Lok Chan et al. reported that the relationship between CT-derived skeletal muscle index (CT-SMI) and chemotherapy-related hematologic toxicity in gastric cancer. They found that low SMI is an independent predictor of hematologic toxicity. Additionally, they observed that patients with low SMI had a significantly shorter overall survival (OS) (17). Tadayoshi Hashimoto et al. found that patients with low psoas muscle index (PMI) had an increased likelihood of experiencing both hematologic and non-hematologic toxicities during preoperative chemotherapy for gastric cancer (31). Research on body composition and hematologic adverse reactions has primarily focused on breast cancer. Both A. L. Wong et al. and Min Kyeong Jang et al. have reported that low muscle mass and excessive body fat mass increase the risk of grade 3 or higher hematologic toxicity during chemotherapy in breast cancer patients (32–33). Although our study did not observe a relationship between body composition and grade 3 hematologic adverse reactions, we found that greater muscle mass during treatment was associated with a reduced risk of grade 2 thrombocytopenia, whereas greater body fat mass increased this risk. M. Bretagne et al. estimated the glomerular filtration rate (GFR) in cancer patients with abnormal body composition and its relationship with carboplatin toxicity. They found that a reduction in lean body mass increased the risk of severe thrombocytopenia (34). To our knowledge, there have been no prior reports on the relationship between body fat mass, total body water, and hematologic toxicity during treatment in gastric cancer patients. The association between increased body fat mass and grade 2 thrombocytopenia observed in our study may have several possible explanations. Increased adiposity may contribute to chronic low-grade inflammation and altered adipokine signaling, which could influence the bone marrow microenvironment and platelet regulation. In addition, chemotherapy dosing based on body surface area may not fully capture interindividual differences in body composition, potentially affecting drug exposure and toxicity. Nevertheless, this finding should be interpreted cautiously, as residual confounding cannot be excluded in this retrospective study.

Most previous studies have assessed body composition using CT-based methods. We acknowledge that, particularly for visceral fat area (VFA), BIA provides an indirect estimate and is less accurate than CT. In the present study, BIA was used because it allowed standardized and repeated assessment of multiple body composition parameters before and after neoadjuvant therapy in routine clinical practice, including muscle mass, body fat mass, total body water, and VFA, without additional radiation exposure. This approach may help identify patients at higher risk of poor treatment tolerance. In addition, BIA has been recognized as a practical tool for body composition assessment in clinical settings. Therefore, our findings regarding VFA should be interpreted with caution, and CT-based assessment would be preferable when precise quantification of visceral adiposity is required (35–36).

It should be noted that although preservation of muscle mass may be clinically important, our study did not directly evaluate exercise interventions. Moreover, patients with gastric cancer undergoing neoadjuvant therapy often have sarcopenia, fatigue, reduced oral intake, and poor physical performance, which may limit their ability to perform conventional resistance training. Therefore, exercise recommendations in this population should be individualized. Low-intensity or rehabilitation-based resistance exercises, together with general physical activity as tolerated, may be more feasible than conventional resistance training in routine practice. Future prospective studies are needed to evaluate the safety, feasibility, and effectiveness of such strategies in this population.

Although our study used multiple body composition parameters (muscle, body fat mass, and total body water) to explore their relationship with the effectiveness and adverse events of neoadjuvant therapy, there are still several limitations. First, this is a retrospective study with a small sample size conducted at a single center, and all data were obtained from patient medical records. Future studies should involve larger sample sizes and prospective designs to explore the relationship between changes in body composition and outcomes of neoadjuvant therapy in gastric cancer. Second, due to the limited sample size, subgroup analyses (e.g., by sex, treatment regimen, and pathological type) and sensitivity analyses were restricted. Therefore, the potential modifying effect of histological subtype on the associations between body composition, reduced RDI, and hematologic toxicity could not be fully evaluated. Third, we did not assess the nutritional status of the patients in this study, which could be a key factor influencing the outcomes of neoadjuvant therapy. In addition, the observed association between increased body fat mass and grade 2 thrombocytopenia may have been influenced by residual confounding, since baseline platelet count, nutritional and inflammatory status, liver or splenic changes, and cumulative drug exposure were not included in the adjustment model. Moreover, because of the retrospective observational design, the temporal and causal relationship between maintenance of muscle mass and treatment tolerance could not be fully disentangled. Fourth, the study participants were all from a Chinese population, which limits the ability to generalize the results to other ethnic groups. In addition, VFA in this study was derived from BIA rather than CT. Because BIA provides only an indirect estimate of visceral adiposity and is less accurate than imaging-based methods, the results related to VFA should be interpreted cautiously. Furthermore, although muscle-related body composition measures were associated with reduced RDI in our study, we did not derive specific clinical cut-off values because the variables were analyzed as continuous measures and the sample size was limited. Future larger prospective studies are needed to establish and validate clinically meaningful thresholds for predicting reduced RDI.

## 5 Conclusion

Our study aimed to use bioelectrical impedance analysis (BIA) to assess the relationship between changes in body composition during neoadjuvant therapy and chemotherapy dose intensity as well as hematologic adverse reactions in gastric cancer patients. We found that during treatment, higher muscle mass and total body water (TBW) were associated with a reduced risk of decreased RDI, while body fat mass showed no significant relationship. Regarding hematologic adverse reactions, a reduction in muscle mass increased the risk of grade 2 thrombocytopenia, whereas an increase in body fat mass was associated with a higher risk. These findings suggest that preserving muscle mass during neoadjuvant therapy may be clinically relevant, although the optimal supportive intervention strategy requires further investigation.

## Data Availability

The original contributions presented in the study are included in the article/Supplementary material, further inquiries can be directed to the corresponding author.
