# Supplementary material for: Body composition–based nutritional status during neoadjuvant chemotherapy and its association with relative dose intensity and hematologic toxicity in patients with gastric cancer
Source: Front Nutr. 2026 Apr 16;13:1753568. doi: 10.3389/fnut.2026.1753568 (PMC13128401; doi:10.3389/fnut.2026.1753568)
Supplement: Supplementary file 1 [file Table_1.docx]

Supplementary Table1：Body Composition Measurements Before and After Neoadjuvant Therapy

| **Body composition** |  | **Pre-treatment** | **Post-treatment** |
| --- | --- | --- | --- |
| **Muscle** |  |  |  |
| SLM (kg) |  | 46.63（7.67） | 46.23（7.67） |
| SMM (kg) |  | 27.98（4.60） | 27.74（4.60） |
| **Adipose tissue** |  |  |  |
| BFM (kg) |  | 12.38（5.97） | 11.29（4.82） |
| VFA (cm^2^) |  | 94.08（41.24） | 84.95（33.47） |
| **Water content** |  |  |  |
| TBW (L) |  | 36.22（6.01） | 35.86（5.99） |

Abbreviations: SLM, muscle mass; BFM,body fat mass; SMM,skeletal muscle mass;

VFA,visceral fat area; TBW,total water content;

Supplement Table2：Body Composition Measurements for Males and Females

| **Body composition** | | **Male** |  | **Female** |
| --- | --- | --- | --- | --- |
| **Muscle** |  |  |  |  |
| SLM change(kg) |  | -0.423（3.767） |  | -0.282（2.544） |
| SMM change(kg) |  | -1.042（3.285） |  | -1.500（1.837） |
| **Adipose tissue** |  |  |  |  |
| BFM change(kg) |  | -0.244（2.252） |  | -0.173（1.491） |
| VFA change(cm^2^) |  | -9.062（26.458） |  | -9.636（10.764） |
| **Water content** |  |  |  |  |
| TBW change(L) |  | -0.374（2.902） |  | -0.282（2.009） |

Abbreviations: SLM, muscle mass; BFM,body fat mass; SMM,skeletal muscle mass;

VFA,visceral fat area; TBW,total water content;

Supplement Table3：Relationship Between Body Composition Changes and Grade 2 Hematological Toxicities

| Hematologic toxicities CTCA（grade2） |  | SLM change  (kg) | p | SMM change  (kg) | p | BFM change  (kg) | p | VFA change  (cm^2^) | p | TBW change  (L) | p |
| --- | --- | --- | --- | --- | --- | --- | --- | --- | --- | --- | --- |
| Anemia |  |  | 0.846 |  | 0.822 |  | 0.371 |  | 0.891 |  | 0.808 |
| Yes |  | -0.53（4.23） |  | -0.32（2.53） |  | -1.58（3.92） |  | -9.72（24.68） |  | -0.48（3.29） |  |
| No |  | -0.36（3.42） |  | -0.20（2.04） |  | -0.92（2.81） |  | -8.91（25.39） |  | -0.32（2.62） |  |
| Neutropenia |  |  | 0.376 |  | 0.368 |  | 0.515 |  | 0.782 |  | 0.365 |
| Yes |  | -0.90（3.47） |  | -0.54（2.07） |  | -1.41（3.95） |  | -10.21（28.84） |  | -0.76（2.75） |  |
| No |  | -0.18（3.71） |  | -0.10（2.21） |  | -0.95（2.72） |  | -8.63（23.26） |  | -0.18（2.83） |  |
| Thrombocytopeia |  |  | 0.044 |  | 0.047 |  | 0.019 |  | 0.022 |  | 0.056 |
| Yes |  | -2.58（4.15） |  | -1.52（2.49） |  | 1.09（2.54） |  | 7.90（20.69） |  | -1.96（3.13） |  |
| No |  | -0.14（3.50） |  | -0.08（2.09） |  | -1.36（3.12） |  | -11.21（24.86） |  | -0.17（2.71） |  |
| Leukopenia |  |  | 0.934 |  | 0.939 |  | 0.229 |  | 0.474 |  | 0.891 |
| Yes |  | -0.35（3.26） |  | -0.20（1.94） |  | -0.39（2.86） |  | -5.77（27.58） |  | -0.29（2.52） |  |
| No |  | -0.42（3.75） |  | -0.25（2.25） |  | -1.32（3.21） |  | -10.19（24.24） |  | -0.39（2.90） |  |

Abbreviations: SLM, muscle mass; BFM,body fat mass; SMM,skeletal muscle mass; VFA,visceral fat area; TBW,total water content;

P values were calculated using t test

Supplement Table4：Relationship Between Body Composition Changes and Grade 3 Hematological Toxicities

| Hematologic toxicities CTCA（grade3） |  | SLM change  (kg) | p | SMM change  (kg) | p | BFM change  (kg) | p | VFA change  (cm^2^) | p | TBW change  (L) | p |
| --- | --- | --- | --- | --- | --- | --- | --- | --- | --- | --- | --- |
| Anemia |  |  | 0.296 |  | 0.303 |  | 0.533 |  | 0.823 |  | 0.305 |
| Yes |  | 0.67（2.92） |  | 0.40（1.73） |  | -1.65（2.26） |  | -10.73（14.24） |  | 0.45（2.31） |  |
| No |  | -0.55（3.71） |  | -0.32（2.22） |  | -1.02（3.25） |  | -8.91（26.25） |  | -0.47（2.86） |  |
| Neutropenia |  |  | 0.692 |  | 0.686 |  | 0.910 |  | 0.510 |  | 0.681 |
| Yes |  | -0.84（3.07） |  | -0.50（1.85） |  | -0.99（2.52） |  | -14.10（32.39） |  | -0.71（2.38） |  |
| No |  | -0.35（3.71） |  | -0.20（2.21） |  | -1.11（3.22） |  | -8.52（24.20） |  | -0.32（2.86） |  |
| Thrombocytopeia |  |  | 0.248 |  | 0.255 |  | 0.776 |  | 0.658 |  | 0.247 |
| Yes |  | -4.60 |  | -2.70 |  | -0.20 |  | 2.00 |  | -3.60 |  |
| No |  | -0.36（3.62） |  | -0.21（2.16） |  | -1.11（3.16） |  | -9.25（25.17） |  | -0.33（2.80） |  |
| Leukopenia |  |  | 0.328 |  | 0.330 |  | 0.382 |  | 0.087 |  | 0.305 |
| Yes |  | -1.96（3.20） |  | -1.16（1.94） |  | -2.30（2.00） |  | -27.80（38.44） |  | -1.62（2.47） |  |
| No |  | -0.32（3.65） |  | -0.18（2.18） |  | -1.03（3.19） |  | -8.06（23.97） |  | -0.29（2.81） |  |

Abbreviations: SLM, muscle mass; BFM,body fat mass; SMM,skeletal muscle mass; VFA,visceral fat area; TBW,total water content;

P values were calculated using t test
